# An Analytical Solution for Saturable Absorption in Pharmacokinetics Models

**DOI:** 10.1007/s11095-022-03455-z

**Published:** 2022-12-21

**Authors:** C.O.S. Sorzano, M.A. Perez-de-la-Cruz Moreno, J.L. Vilas

**Affiliations:** 1 National Center of Biotechnology, CSIC., Madrid, Spain; 2 Kinestat Pharma, Madrid, Spain

**Keywords:** absorption model, Hill kinetics, Pharmacokinetics, saturable absorption

## Abstract

**Objective:**

The first-order absorption is a common model used in Pharmacokinetics. The absorption of some drugs follows carrier mediated transport. It has been proposed that the amount of drug available may saturate the transport mechanism resulting in an absorption slower than the one predicted by the first-order model. Saturable absorption has been modeled at the differential equation level by substituting the constant rate absorption by a Hill kinetics absorption. However, its exact solution is so far unknown. The goal of this is to know the exact solution of different Hill kinetic absorption models.

**Methods:**

We start defining different absorption models and increasing then their complexity. The simplest case is the first-order absorption model and the most complex will be a generalized Hill kinetic absorption model. The differential equation of each model is integrated.

**Results:**

The complexity of the models their solutions may be not expressed in a close-form, or in term of elementary functions. We obtain and discuss the exact solutions of the different Hill kinetics absorption models. To do that, the solutions are studied according to the possible values of the free parameters of the models. We show the differences between models through simulations.

**Conclusions:**

The knowledge of closed-form solutions allows to illustrate the differences between the different absorption models and minimizes the errors of numerical integration.

## Introduction

Pharmacokinetics is the field of research that studies the time evolution of the concentration of a drug within the organism, in particular, its absorption, distribution, metabolism, and excretion (ADME). This evolution can be mathematically described by using differential equations. Drug absorption obviously plays a central role as a driver (input) of the system [1]. The shape of the absorption curve over time can be given analytically or by a differential equation that it must fulfill. In the second case, there might not be a closed-form solution so that it has always to be treated numerically, which hinders understanding its properties and prevents fast integration. This is the case of paliperidone palmitate, used for the treatment of schizophrenia [2]. Its absorption profile was described by writing the differential equation it should fulfill. It was similar to a first-order absorption, but the absorption constant was replaced by a function that depends on the amount of drug available, very much in the spirit of Hill’s model [3]. This modification was introduced to account for the saturation observed during absorption (when the concentration of the available drug is high, cell transporters are saturated and cannot intake an amount that is proportional to the amount available). Saturable absorption has also been described for a broad variety of drugs, some examples are: ascorbic acid [4, 5], cefatrizine [6], metformin [7, 8], cefuroxime axetil [9, 10], and monoclonal antibodies [11, 12].

However, this modification of the differential equation made it non-linear and its exact solution has remained unknown till now. In this study, we provide an exact solution for saturated absorption. Having an analytical closed-form (exact solution), reduces the uncertainty in the estimation of the absorption and facilitates the analysis of its properties, fast numerical fitting of the absorption constants, and fast simulation. First, we introduce the absorption with the classical first-linear-order absorption model. This linear equation has no free parameters (parameter that generalizes the model), and it is only regulated by the absorption constant. Then, we study the Hill’s model, which has a free parameter the Hill’s exponent. Generally it is not possible to obtain the exact solution of the Hill’s model due to the Hill constant. For this reason we modified the Hill’s model by introducing two free parameters or also called Hill exponents. Although the modification introduced to account for the saturation is inspired by Hill’s equation, it is not exactly equal. We, then, present Hill’s model to put the modification in context and we show the exact solutions of the modified Hill’s model used in [2]. We show that the model proposed generalizes Michaelis-Menten kinetics.

Finally, we illustrate the differences in the time profile of first-order absorption and their saturated counterparts. Saturable absorption is an effect that can happen in all administration routes. For example, in the list above Vitamin C and metformin are given orally, cefatrizine and some antibodies intravenously, and some other antibodies are administered subcutaneously. Irrespective of the administration route, the amount available for absorption will decrease following the differential equations described in this paper (assuming that they are the right model for the compound in question). In this way, our simulations below do not correspond to any specific administration route. On the contrary, they describe the amount of compound available at any given time when the absorption mechanism is saturated.

## Methods

### First-Order Absorption Model

In the standard first-order absorption model, the amount, *A*(*t*), remaining after a time *t* can be described by the differential equation [13, Chap. 6]

1 $$ \frac{dA}{dt}=-K_{a} A $$

where *K*_*a*_ is the absorption rate constant and *A*(0) = *A*_0_ the initial amount given as dose. The solution of this process is the well-known first-order absorption whose analytical solution is

2 $$ A(t)=A_{0} \exp(-K_{a} t). $$

This linear differential (1) is a widely used model. It should be noted, however, that recent studies demonstrate that oral drug absorption is completed in finite time [14–19]

### Hill’s Model

Hill’s model proposes a temporal evolution for the absorption as

3 $$ \begin{array}{rcl} \frac{dA}{dt}&=&-K_{a,max} \frac{A^{\gamma}}{A^{\gamma}+A_{50}^{\gamma}} \\ &=&-\frac{K_{a,max}A^{\gamma-1}}{A^{\gamma}+A_{50}^{\gamma}}A \end{array} $$

where *γ* is the Hill exponent (free parameter), *K*_*a*,*max*_ is the maximum absorption rate, and *A*_50_ the amount at which the absorption rate is at half of its maximum. This differential equation is similar to the first-order absorption, but with an absorption coefficient that depends on the amount available, note that $K_{a}=\frac {K_{a,max}A^{\gamma -1}}{A^{\gamma }+A_{50}^{\gamma }}$. The absorption kinetics governed by a time-dependent coefficient was also previously studied [20–22]. The Eq. 3 is separable and therefore it can be integrated as

4 $$ {\int}_{A_{0}}^{A}\left( 1+\frac{A_{50}^{\gamma}}{A^{\gamma}}\right)dA = - {{\int}_{0}^{t}}K_{a,max}dt , $$

and therefore

5 $$ \begin{array}{@{}rcl@{}} A+\frac{1}{1-\gamma}\frac{A_{50}^{\gamma}}{A^{\gamma-1}} &=& A_{0} + \frac{1}{1-\gamma}\frac{A_{50}^{\gamma}}{A_{0}^{\gamma-1}} - K_{a,max}t. \quad if  \gamma\neq 1 \end{array} $$

6 $$ \begin{array}{@{}rcl@{}} A+A_{50}\ln|A| &=& K_{1}- K_{a,max}t. \quad if  \gamma = 1 \end{array} $$

where $K_{1} = A_{0} + A_{50}\ln |A_{0}|$.

Unfortunately, it is not possible to obtain a closed-form for the amount available *A*, in the most general case, *γ*≠ 1. The case *γ* = 1 can be analytically solved, as shown in Case B of next section.

### Modification of Hill’s Model

As consequence of the lack of closed-form for *A*, it has to be solved numerically from Eq. 5. However, this problem can be alleviated if we decouple the two exponents of Eq. 3:

7 $$ \frac{dA}{dt}=-K_{a,max}\frac{A^{\gamma_{1}}}{A^{\gamma_{2}}+A_{50}^{\gamma_{2}}}, $$

where *γ*_1_ and *γ*_2_ are the Hill exponents. Note that when *γ*_1_ = *γ*_2_, then Eq. 7 results in the Hill’s model (3). We now integrate (7)

8 $$ {\int}_{A_{0}}^{A}\frac{A^{\gamma_{2}}+A_{50}^{\gamma_{2}}}{A^{\gamma_{1}}}dA + {{\int}_{0}^{t}}K_{a,max}dt = 0, $$

resulting in

9 $$ \begin{cases} (a) \frac{1}{\gamma_{2}-\gamma_{1}+1}A^{\gamma_{2}-\gamma_{1} + 1} + \frac{A_{50}^{\gamma_{2}}}{1-\gamma_{1}} A^{1-\gamma_{1}} = Q_{a}(\gamma_{1},\gamma_{2}, t) & \text{if}  \gamma_{1}\neq 1, \gamma_{2}-\gamma_{1}\neq1\\ (b) \frac{1}{\gamma_{2}}A^{\gamma_{2}} + A_{50}^{\gamma_{2}}\ln|A| = Q_{b}(\gamma_{2}, t) & \text{if}  \gamma_{1}= 1, \gamma_{2}\neq0,\\ (c) \ln|A| = \frac{1}{2}Q_{c}(t) & \text{if}  \gamma_{1}= 1, \gamma_{2}=0,\\ (d) \ln|A| - \frac{A_{50}^{\gamma_{2}}}{\gamma_{2}} A^{-\gamma_{2}} = Q_{d}(\gamma_{2}, t) & \text{if}  \gamma_{1}\neq 1, \gamma_{2}-\gamma_{1}=-1 \text{with } \gamma_{2}\neq0. \end{cases} $$

where,

$$ \begin{array}{@{}rcl@{}} Q_{a}(\gamma_{1},\gamma_{2}, t) &=& \frac{1}{\gamma_{2}-\gamma_{1}+1}A_{0}^{\gamma_{2}-\gamma_{1} + 1} + \frac{A_{50}^{\gamma_{2}}}{1-\gamma_{1}} A_{0}^{1-\gamma_{1}} \\&&- K_{a,max}t,\\ Q_{b}(\gamma_{2}, t) &=& \frac{A_{0}^{\gamma_{2}}}{\gamma_{2}} + A_{50}^{\gamma_{2}}\ln|A_{0}|- K_{a,max}t,\\ Q_{c}(t) &=& \ln|A_{0}| - K_{a,max}t,\\ Q_{d}(\gamma_{2}, t) &=& \ln|A_{0}| - \frac{A_{50}^{\gamma_{2}}}{\gamma_{2}} A_{0}^{-\gamma_{2}} - K_{a,max}t. \end{array} $$

It is noteworthy that the absolute value can be omitted, because negative absorption lacks pharmacological meaning. However, it has been kept to be rigorous.

#### Case A: *γ*_1_≠ 1,*γ*_2_ − *γ*_1_≠ 1

This is the most general case. Unfortunately, the amount A cannot be expressed in simple terms. Even if *γ*_1_ and *γ*_2_ are integers, the Abel-Ruffini theorems [23] establish that explicit solutions cannot be obtained unless the resulting equation has degree lesser than five. Thus, this case will not be treated. However, the other three possibilities in Eq. 9 will be analyzed.

#### Case B: *γ*_1_ = 1,*γ*_2_≠ 0

A good example of this modification case is the non-linear differential equation proposed by Gopal et al. [2] for paliperidone palmitate. To obtain an analytical expression is necessary to rewrite (9)b, as

10 $$ |A|^{\gamma_{2}}e^{\frac{|A|^{\gamma_{2}}}{A_{50}^{\gamma_{2}}}}=e^{\frac{\gamma_{2}Q_{b}}{A_{50}^{\gamma_{2}}}}. $$

The amount of drug A can be expressed in a close-form by means of the W-Lambert function [24]. This function is defined as the inverse function of *f*(*z*) = *ze*^*z*^, such as *z* = *W*(*f*). Just dividing both terms by $A_{50}^{\gamma _{2}}$ and applying the W-Lambert function results in

11 $$ A= A_{50}W\left( \frac{1}{A_{50}^{\gamma_{2}}}e^{\frac{\gamma_{2}Q_{b}}{A_{50}^{\gamma_{2}}}}\right)^{-\gamma_{2}}. $$

where *W*(*x*) is the main branch of Lambert’s *W* function and it is available in most advanced scientific computing languages (Matlab, Python scipy, C++ Boost, C GNU Scientific Library).

The Michaelis-Menten kinetics is a particular solution of this family of abortion models for which *γ*_1_ = *γ*_2_ = 1. In this case the solution becomes simpler as

12 $$ A= A_{50}W\left( \frac{1}{A_{50}}e^{\frac{Q_{b}}{A_{50}}}\right). $$

or alternatively, using the Wright function

13 $$ A(t) = A_{50}\omega\left( \gamma\frac{K_{1} - K_{a,max}t}{A_{50}^{\gamma}}\right)^{1/\gamma}. $$

#### Case C: Standard First-Order Model *γ*_1_ = 1,*γ*_2_ = 0

The modified Hill’s equation results in the standard first-order absorption model, it is $\frac {dA}{dt}=-K_{a}A$, with solution *A*(*t*) = *A*_0_*exp*(−*K*_*a*_*t*) (seen above). Note that, if *γ*_1_ = 1, and *γ*_2_ = 0, then $\frac {dA}{dt}=-K_{a,max}\frac {A}{2}$, therefore, *K*_*a*_ = *K*_*a*,*max*_/2. Thus, the solution derived from the modified Hill’s model in Eq. 9c recovers the original result of the standard first-order model.

#### Case D: *γ*_1_≠ 1,*γ*_2_ − *γ*_1_ = − 1 with *γ*_2_≠ 0

In this case, the result of the integration resulted in

14 $$ \ln|A|- \frac{A_{50}^{\gamma_{2}}}{\gamma_{2}}A^{-\gamma_{2}} = Q_{d} $$

or alternatively, multiplying by − *γ*_2_ and taking the exponential in both terms

15 $$ A^{-\gamma_{2}}e^{A_{50}^{\gamma_{2}}A^{-\gamma_{2}}}=e^{-\gamma_{2}Q_{d}}. $$

Now it is possible to make use of the W-Lambert function [24] to isolate the amount A as follows

16 $$ A_{50}^{\gamma_{2}}A^{-\gamma_{2}} = W\left( A_{50}^{\gamma_{2}}e^{-\gamma_{2}Q_{d}}\right) $$

and therefore

17 $$ A = \frac{A_{50}}{W\left( A_{50}^{\gamma_{2}}e^{\gamma_{2}Q_{d}}\right)^{1/\gamma_{2}}} $$

## Simulations

**Fig. 1:**
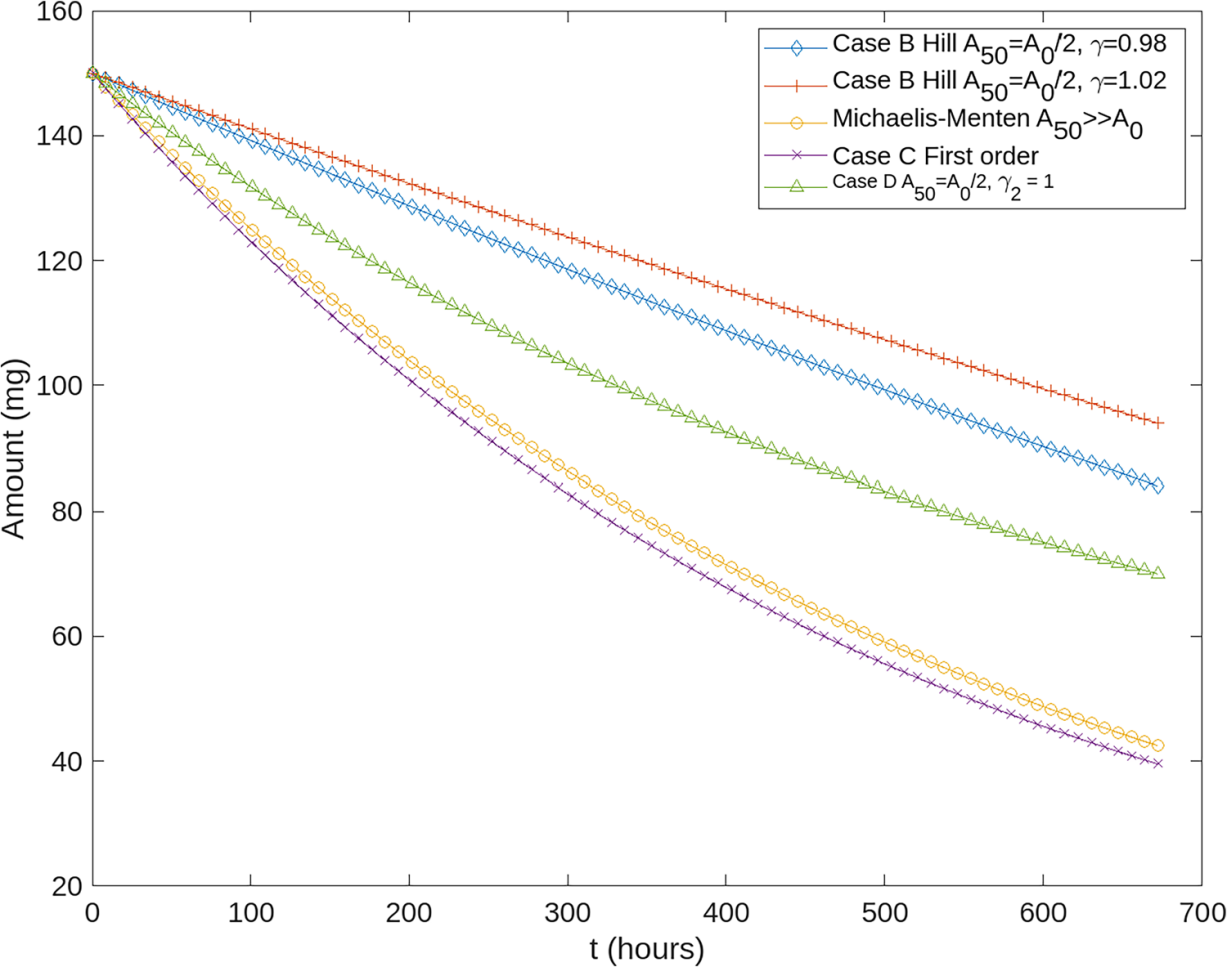
Example of the remaining amount of drug after time *t*. Case C: The first order absorption has an absorption constant *K*_*a*_ = 1/3 (h^− 1^) and *A*_0_ = 150 (mg.). Michaelis-Menten has *A*_50_ = 10*A*_0_ and *K*_*a*,*max*_ = *A*_50_*K*_*a*_. Case B: Two Hill kinetics have been simulated with *A*_50_ = *A*_0_/2, *K*_*a*,*max*_ = *A*_50_*K*_*a*_ and *γ* = 0.98 and *γ* = 1.02 (note that *γ* = 1 gives the special case of the Michaelis-Menten kinetics, values of gamma slightly below and above *γ* = 1 have been chosen to illustrate their difference with respect to Michaelis-Menten, and how small deviations from 1 imply significant differences in the absorption profiles). Case D: *A*_50_ = *A*_0_/2, *K*_*a*,*max*_ = *K*_*a*_, *γ*_2_ = 1.

For comparison purposes, we have simulated the curves for the remaining amount of drug after time *t* (in hours) for a first-order absorption, an equivalent Michaelis-Menten (in which the saturation almost does not apply), and two saturated extended Hill kinetics with slightly different *γ*_2_ values to illustrate their effect. These simulations correspond to the cases B, C, and D. Figure 1 shows the results of the simulations, observing a close behaviour between the different models B and C, and therefore validating our theoretical results. It must be highlighted that the saturation mechanism does not apply if *A*_50_ ≫ *A*_0_. If $K_{a,max}=K_{a} A_{50}^{\gamma }$, then the saturated absorption solution is almost the same as the first-order absorption with constant *K*_*a*_.

All curves show a general decay over time, as it should as the amount of remaining compound available for absorption decreases over time. The parameters of the simulation have been chosen so that they all exhibit similar behavior. However, the specific local slope of the various curves differ according to the specifications of their respective differential equations. These differences can make the pharmacokinetic fitting better or worse depending on whether the underlying absorption model truly represents reality or not. In this regard, having access to the analytical form of the absorption profile allows a much quicker evaluation of the parameters proposed by the optimizer used during the fitting.

## Conclusion

In this work, several saturable absorption models based on the Hill’s model and its modification have been proposed. To our knowledge, the pharmacological models that make use of the Hill’s equation make use of numerical methods to find the solution. In contrast, in this study the exact solutions in their closed-form are obtained. To do that, we started by modifying the Hill’s equations, by defining two exponents, one for the numerator and other for denominator respectively. This simple modifications allows different approaches to obtain an exact integration of the modified Hill’s equation, as well as, generalizes the absorption model. In particular, our generalized model recovers the first order standard model, and we analyzed the cases of the Michaelis-Menten equation and a family of Hill’s equations with *γ*_1_ = 1 and *γ*_2_ = *γ* (where *γ* is a free parameter). The knowledge of the exact solution opens a broad variety of possibilities in the analysis of pharmacological data, enhancing the understanding of the model and increasing the reliability of the data fitting.
